# Silk Fibroin Biomaterials and Their Beneficial Role in Skin Wound Healing

**DOI:** 10.3390/biom12121852

**Published:** 2022-12-12

**Authors:** Łukasz Mazurek, Mateusz Szudzik, Mateusz Rybka, Marek Konop

**Affiliations:** Department of Experimental Physiology and Pathophysiology, Laboratory of Center for Preclinical Research, Medical University of Warsaw, 02-106 Warsaw, Poland

**Keywords:** silk fibroin, silk biomaterials, wound healing, skin wounds, burn wounds, diabetic wounds

## Abstract

The skin, acting as the outer protection of the human body, is most vulnerable to injury. Wound healing can often be impaired, leading to chronic, hard-to-heal wounds. For this reason, searching for the most effective dressings that can significantly enhance the wound healing process is necessary. In this regard, silk fibroin, a protein derived from silk fibres that has excellent properties, is noteworthy. Silk fibroin is highly biocompatible and biodegradable. It can easily make various dressings, which can be loaded with additional substances to improve healing. Dressings based on silk fibroin have anti-inflammatory, pro-angiogenic properties and significantly accelerate skin wound healing, even compared to commercially available wound dressings. Animal studies confirm the beneficial influence of silk fibroin in wound healing. Clinical research focusing on fibroin dressings is also promising. These properties make silk fibroin a remarkable natural material for creating innovative, simple, and effective dressings for skin wound healing. In this review, we summarise the application of silk fibroin biomaterials as wound dressings in full-thickness, burn, and diabetic wounds in preclinical and clinical settings.

## 1. Introduction

The skin is the largest human organ. It forms an outer barrier to protect against the external environment and also protects against the loss of water, electrolytes, and proteins from the human body, maintaining homeostasis [1,2,3,4]. 

The skin is constantly exposed to potential damage. Depending on the size of the injury, skin defects can leave varying degrees of scarring. Crucially, for cosmetic and psychological reasons scars can impair patients’ quality of life [5]. Skin damage initiates a series of processes leading to its healing. Many local factors such as infection or systemic factors like diabetes, obesity, alcohol, smoking, and malnutrition, impair wound healing. These conditions can lead to chronic and non-healing wounds [6], a major medical problem that requires new therapeutic options. Due to this, scientists worldwide seek an ideal wound dressing that accelerates impaired wound healing.

Several natural materials like collagen [7,8,9], gelatine [10,11,12], keratin [13,14,15], and silk [16,17,18] have gained much attention in tissue engineering and regenerative medicine as potential skin wound dressings. Especially noteworthy are wound dressings based on silk fibroin (SF), characterized by good tissue biocompatibility and biodegradability. It is also possible to enrich these biomaterials with additional substances [19] that can enhance the therapeutic process.

Silk fibres are naturally produced protein fibres most associated with silkworms (*Bombyx mori*). They are also produced by spiders, scorpions, flies, and mites [20]. Currently, only silkworm silk has large-scale use. Silk fibres are commercially used in many areas of the economy, and they play an essential role in the textile industry. However, they are also used in medical applications such as sutures or skin wound dressings [20,21,22,23]. Also noteworthy is that silk was used during the COVID-19 pandemic by being included in protective fabric masks. Konda et al. [24] investigated the effectiveness of the most common fabric mask components and found that silk could be an influential element in multilayer cover masks.

Silkworm silk is insoluble in water and consists of two types of proteins: fibroin proteins, which are the main structural element accounting for about 70% of the cocoon, and sericin proteins, which bond the fibroin filaments to each other and account for about 30% [25,26,27]. SF is a widely used tissue-biocompatible protein, especially in biomedical applications [16], and was admitted as a biomaterial by the U.S. Food and Drug Administration (FDA) in 1993 [28]. On the other hand, silk sericin has been traditionally suggested to have immunogenic effects and to induce an inflammatory response. Therefore, removing the sericin protein from the cocoon is essential to increasing the biocompatibility and additional biodegradability of silk fibres [20]. However, it is now thought that pure silk sericin does not increase inflammation. Jiao et al. [29] showed that sericin can initiate low inflammatory cell infiltration, similar to fibroin, and minor allergenic and immunogenic reactions. Furthermore, silk sericin can also be used in regenerative medicine and wound healing [30,31,32] or even in cosmetics and other pharmaceutical uses [27,33]. However, SF biomaterials are predominant in medical applications over silk sericin.

In this review, we describe recent studies of the effect of the properties of different kinds of SF-based dressings on the healing process of full-thickness skin wounds, diabetic wounds, and burn wounds.

## 2. The Physiology of Skin Wound Healing

Wound healing is a complex physiological process that occurs immediately after the loss of skin integrity. This process progresses in subsequent steps and usually consists of four continuous and overlapping phases: haemostasis, inflammation, proliferation, and tissue remodelling [34].

Haemostasis is the first stage of wound healing, which can take up to two days. As a result of trauma, the mechanisms of haemostasis are activated. The main factors stimulating the clotting mechanism and thus initiating the haemostatic process include vasoconstriction, platelet aggregation, and fibrin deposition. The clot formed consists of aggregated platelets and fibrin mesh with embedded blood cells. It prevents further loss of fluid and electrolytes from the wound and provides a primary line of defence against pathogens from the external environment. Aggregated platelets secrete factors such as platelet-derived growth factor (PDGF) and transforming growth factor β (TGFβ) and attract various cell types (fibroblasts and inflammatory cells) while initiating the healing process [6,35,36].

The main features of inflammation include pain, elevated temperature, redness, and tissue swelling. The inflammation is characterized by the migration of inflammatory cells to the site of injury [37]. Numerous vasoactive (e.g., serotonin, bradykinin, prostaglandins, and histamine) and chemotactic factors are secreted during this process. Tissue-localised immune cells (mast cells, T lymphocytes (δ, γ), and Langerhans cells) are activated, causing the release of chemokines (including extracellular matrix protein fragments, TGF-β, PDGF, and monocyte chemoattractant protein-1 (MCP-1)) and cytokines (including colony-stimulating factor 1 (CSF-1), tumour necrosis factor α (TNF-α), transforming growth factor alpha (TGF-α), and insulin-like growth factor 1 (IGF-1)) [38,39]. Afterward, macrophages residing in the tissue initiate a local inflammatory response, leading to the influx of large numbers of neutrophils into the wound. Neutrophils are tasked with cleansing the wound of foreign bodies, damaged and dead cells, and bacteria. After fulfilling their role, neutrophils are removed as a scab or are phagocytosed by macrophages. Like platelets, macrophages secrete cytokines, including growth factors, which are involved in migration, proliferation, and vascular growth within the connective tissue of the wound [37,38,39,40,41].

Briefly, the proliferative phase starts about three days after injury and lasts 2 to 4 weeks. It is characterised by the migration of fibroblasts and vascular endothelial cells, deposition of extracellular matrix, and granulation tissue formation. Fibroblasts and keratinocytes play a crucial role in this stage. Increased expression of cell membrane integrin receptors that bind fibronectin and fibrin into the temporary wound matrix contributes to fibroblast migration. Factors stimulating fibroblast migration include fibronectin, PDGF, TGF-β, and epidermal growth factor (EGF) [36,37,38,39,40,41]. The early stages of wound healing are also characterised by hypoxia. Hypoxia activates the hypoxia-inducible factor (HIF-1α), which then stimulates the expression of the vascular endothelial growth factor (VEGF-A). It results in active endothelial cell proliferation, increased wound capillary density, and blood flow restoration [36,42,43]. Secretion of matrix metalloproteinases (MMPs) is required to facilitate cell migration within the scab and scar. The final stage of the proliferation phase is the formation of granulation tissue. The newly formed connective tissue (granulation tissue) replaces the clot made of fibrin and fibronectin. The predominant cell type in granulation tissue is fibroblasts, which produce significant amounts of fibronectin, hyaluronic acid, and collagen types I and III [44,45]. They are essential in the later stages of the healing process. Next, fibroblasts transform into myofibroblasts, which are involved in wound contraction [46].

Wound contraction begins around 4 to 5 days after injury and continues for another two weeks. However, this process is longer in wounds that are still open after two weeks of healing. In an open wound, the shrinking process is evident as the edges of the wound are drawn together. Shrinking the surgical wound results in a shorter scar, and thus the scar is less visible. The shape of the wound affects the shrinking process. Square wounds shrink faster than circular wounds [47,48]. The rate of wound contraction varies depending on the anatomical location and averages 0.6-0.7 mm per day. Myofibroblasts have a contractile capacity, which results in a reduction of the wound area. They determine the wound contraction process at the wound edges. These cells appear at the injury site between 4 and 6 days and are present in the reconstructed tissue for another 2–3 weeks [49,50].

Scar remodelling is the final step in the normal healing process, which begins around day 21 after injury at various times and places within the injured tissue. Macrophages and fibroblasts activate growth factors associated with the extracellular matrix (ECM) and MMPs. The collagen synthesis rate decreases and converges with the rate of collagen degradation. Inhibition of collagen synthesis occurs under the influence of interferon gamma (IFN-γ), TNF, and the collagen matrix. The characteristic stage of this phase is the replacement of type III collagen (synthesized in the first days after injury) with more stable type I collagen until the balance typical for healthy tissue is achieved (I:III = 4:1) [46,51,52].

## 3. Preparation of SF-Based Dressings

In recent years, there has been a growing search for a biomaterial matrix that could support the development of living and biologically active tissue, both in vivo and in vitro. The Food and Drug Administration (FDA) has approved SF as a biomaterial that can be successfully used in tissue engineering due to its unique properties and complex structure [53].

Regardless of the SF scaffold preparation method, the raw silk cocoon must first undergo degumming to eliminate sericin. This process is carried out by boiling raw silk cocoons in sodium carbonate (Na_2_CO_3_). Careful control of the time of this process and Na_2_CO_3_ concentration is essential, given the risk of adverse effects on fibroin. The fibres are then rinsed with pure water and dried overnight [54,55,56]. The most common approach for obtaining silk-based materials is to dissolve SF filaments in a pure fibroin solution and then regenerate the SF into various material formats [57]. In order to dissolve SF, different types of solvents are used, such as an aqueous solution of lithium bromide (LiBr), Ajisawa reagent (CaCl_2_:EtOH:H_2_O in a molar ratio of 1:8:2), an aqueous solution of calcium nitrate (Ca(NO_3_)_2_), an aqueous solution of lithium thiocyanate (LiSCN), N-methylmorpholine oxide, hexafluoroisopropanol (HFIP), or ionic liquids [56,58]. Next, the solution is purified by dialysis against pure water to obtain an aqueous fibroin solution. Finally, it can be used as a substrate for production of biomaterials and is able to be stored for a long time [56].

**Figure 1:**
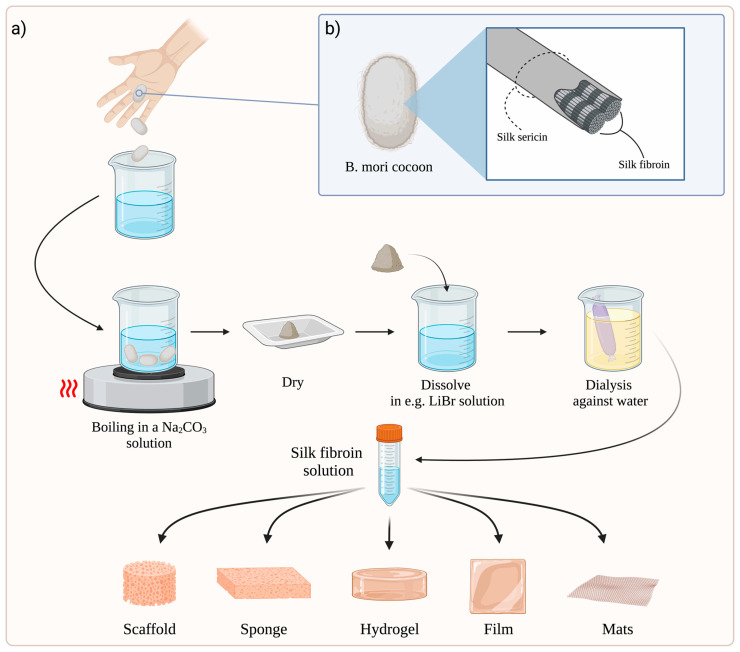
(**a**) General schematic process for obtaining various types of dressings based on SF. The figure was created based on Thurber et al. [69] and refs. [54,55,56,57,58,70]. (**b**) General scheme of silk fibre structure. Fibroin is bonded and held together by sericin [25,26,27]. Abbreviations: B. mori—Bombyx mori; Na_2_CO_3_—sodium carbonate; LiBr—lithium bromide. Created with BioRender.com (accessed on 10 November 2022).

The SF solution is generally used to produce scaffolds [59,60], sponges [61,62], hydrogels [63,64], films [65,66], and electrospun mats [67,68], which have applications in skin wound healing (Figure 1a).

## 4. Molecular Mechanisms of SF Action

SF protein is involved in every phase of wound healing on the physiological and molecular levels [16]. There is increased inflammatory cell infiltration, production of inflammatory cytokines, cell proliferation, and wound remodelling through the activation of many cellular signalling pathways. However, the role of SF in regulating particular molecular pathways is not well known and has prompted scientists to explore the molecular properties of this dressing [23].

So far, most scientific work has focused on the in vitro study of fibroin properties. However, several studies have tried to unravel the molecular mechanism underlying SF’s action in vivo. 

In mice studies conducted by Yun et al. [71] on memory and learning, enzymatic SF hydrolysate (FEH) was shown to protect mice from scopolamine-induced memory and learning impairment. Moreover, FEH treatment increased acetylcholine (ACh) and brain-derived neurotrophic factor (BDNF) levels and phosphorylated cAMP response-element binding protein (p-CREB) by stimulating p-PI3K/p-AKT/mTOR/PSD95 (p-PI3K—phosphorylated phosphoinositide 3-kinase, p-AKT—phosphorylated protein kinase B, mTOR—mammalian target of rapamycin, PSD95—postsynaptic density protein-95), extracellular signal-regulated kinase (ERK), and Ca^2+^/calmodulin-dependent protein kinase II (CaMKII) pathways in brain hippocampal tissue. It is also relevant that FEH decreased interleukin-1β (IL-1β), TNF-α, and interleukin-6 (IL-6) cytokines. It is an important report, showing the neuroprotective properties of SF hydrolysate. Its inflammation-reducing properties appear to be an essential finding for the wound healing process.

Park et al. [72] performed in vitro and in vivo studies using a fibroblast cell line isolated from a mouse NIH/Swiss embryo (NIH/3T3) and Sprague–Dawley rats. Their results showed an increased expression of nuclear factor κ-light-chain-enhancer of activated B cells (NF-kB) signalling pathway genes during SF treatment. They also demonstrated higher protein levels of vimentin, fibronectin, cyclin D1, and VEGF after SF treatment, which are the target proteins of the NF-kB signalling pathway. Moreover, this molecular pathway is indispensable in wound healing and affects cell proliferation, adhesion, inflammation, and elimination of reactive oxygen species [73,74,75]. 

Another study, performed by Chou et al. [76], tested the properties of SF in vitro and in vivo. The authors showed that SF in the form of β-sheet promoted the expression of fibronectin, type III collagen, matrix metalloproteinase-12 (MMP-12), and integrin β1 in the granulation tissue of burn wound model rats and human dermal fibroblasts (HDFs).

In an in vitro study in a human spontaneously immortalised keratinocyte cell line (HaCaT) and human mammary gland cells (MDA-MB-231), SF upregulated three different pathways: mitogen-activated protein kinase kinase (MEK1), phosphoinositide 3-kinase (PI3K) and c-Jun N-terminal kinase (JNK), which led to activation of cell migration and induced c-Jun expression through downstream mechanisms [77].

A study of bone marrow-derived human mesenchymal stem cells (hMSCs) regarding bone regeneration demonstrated that an SF scaffold increases runt-related transcription factor 2 (Runx2), bone morphogenetic proteins (BMPs), collagen, osterix, osteopontin, and osteocalcin biomarkers of differentiation into osteoblasts compared to traditionally used ceramic-based scaffolds [78]. This research indicates the potential for the broad application of SF in regenerative medicine.

An interesting study conducted by Naserzadeh et al. [79] revealed the toxic effect of SF. Application of silk nanoparticles on isolated fibroblasts and human umbilical vein endothelial cells (HUVECs) indicated mitochondrial dysfunction, reactive oxygen species (ROS) production, and lipid peroxidation, connected with cytochrome c release. However, the authors did not indicate a clear effect of SF on a specific molecular pathway. The toxicity of SF should be investigated further and in more detail to understand the exact mechanism of action.

**Figure 2:**
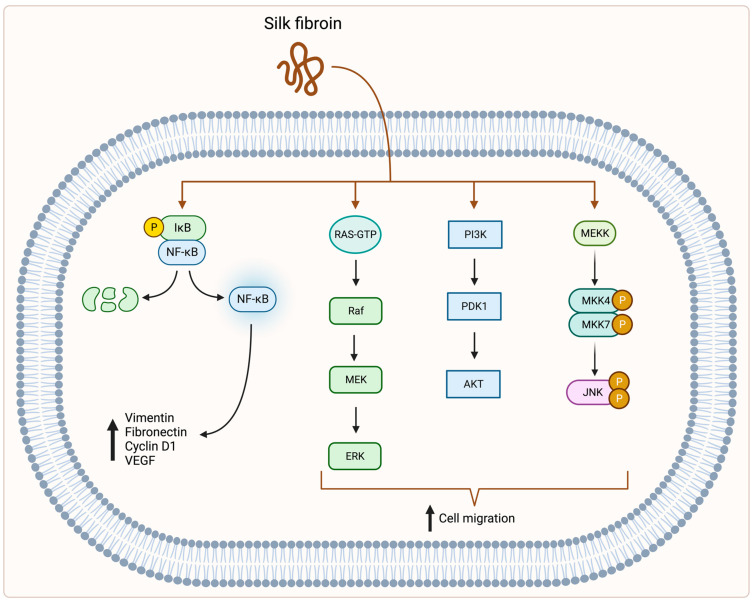
SF stimulates the expression of NF-kB signalling pathway genes, which leads to higher expression of vimentin, fibronectin, cyclin D1, and VEGF [72]. SF also stimulates MEK, PI3K, and JNK pathways, which leads to increased cell migration [77]. Abbreviations: IκB—inhibitor of NF-κB; NF-κB—nuclear factor κ-light-chain-enhancer of activated B cells; RAS—RAS protein; GTP—guanosine-5’-triphosphate; Raf—rapidly accelerated fibrosarcoma; MEK—mitogen-activated protein (MAP) kinase kinase; ERK—extracellular signal-regulated kinase; PI3K—phosphoinositide 3-kinase; PDK1—phosphoinositide-dependent kinase-1; AKT—protein kinase B; MEKK—mitogen-activated protein (MAP) kinase kinase kinase; MKK4/MKK7—MAPK kinases; JNK—c-Jun N-terminal kinase; P—phosphorylated. Created with BioRender.com (accessed on 14 November 2022).

All the above-mentioned studies show general molecular mechanisms affected by SF (Figure 2). Such basic knowledge provides opportunities for researchers and is a motivation for more detailed studies, including animal models. Given the favourable properties of SF in wound healing, it is certainly worth exploring the molecular basis of fibroin action in a much more complex way.

## 5. Antibacterial Properties of Silk Fibres

Antimicrobial properties are an expected feature of dressings used in skin wound healing. Silk fibres are characterized by a lack of evident antimicrobial properties. Moreover, they provide a good underlayer for bacterial growth. Various strains of bacteria form a biofilm on the surface of silk fibres [80,81,82]. Seves et al. [81] showed that bacterial growth is more notable on raw silk containing fibroin and sericin proteins than on degummed silk consisting of fibroin alone. Therefore, taking advantage of the excellent biomedical properties of silk fibres [20,21,23,70], attempts were made to increase the antimicrobial activity of silk dressings by enhancing them with various types of substances, such as antibiotics [83,84], Ag nanoparticles (NPs) [85,86], or polymers such as chitosan [87,88]. Such procedures increased the efficiency of wound treatment, preventing infection of the wounded skin that could worsen and prolong the healing process [89]. 

## 6. Properties of SF Biomaterials in Skin Wound Healing

### 6.1. The Role of SF-Based Dressings in the Treatment of Full-Thickness Skin Wounds

In recent years, studies of SF-based dressings have presented promising results. Researchers have used various forms of fibroin dressings and different modifications in many experimental models. Recent research focusing on the effects of SF-based dressings on skin wound healing is described below.

Guo et al. [59] examined two kinds of SF scaffolds prepared by an electric field—random SF scaffolds (RSSs) and aligned SF scaffolds (ASSs)—and their impact on vascularisation and wound healing in female Sprague–Dawley rats. In vivo results showed that both types of dressings promote epithelialisation in the full-thickness wound model. During the experiment, wound repair was consistently faster in the ASS group than in the RSS group and was significant on day 14 (*p* ≤ 0.01). However, both types of dressings significantly accelerated wound healing compared to the control (untreated) group. Furthermore, both materials promoted neovascularization in the wounds, which is essential for effective wound healing.

In another study, Zhang et al. [90] compared two different electrospun SF membranes with a control group treated with commercial wound dressing (3M^TM^ Tegaderm^TM^). An electrospun SF membrane was modified by poly-dopamine (PESF), and the second SF dressing did not have any modification (ESF). The full-thickness skin wound models were prepared on Sprague–Dawley rats. The authors showed that all dressings effectively enhanced wound healing. However, they revealed that the poly-dopamine-modified SF dressing was significantly (*p* < 0.001) superior to the others during the healing process. Furthermore, wounds treated with the two types of SF membranes had a smaller inflammatory infiltration on the third day post-surgery than the control group. Complete re-epithelialization was observed in all groups on the fourteenth day. The most effective skin regeneration was observed in the poly-dopamine-modified SF dressing group. This study showed that SF membrane modification can improve wound treatment potential and can be superior to commercially available dressings.

Notably, the diameter of the SF fibres also plays a crucial role in wound healing efficiency. Hodgkinson et al. [60] analysed SF scaffolds with different fibre diameters from 256 ± 30 to 1214 ± 321 nm. In vitro studies with primary human dermal fibroblasts (PHDFs) revealed that the proliferation of PHDFs was the highest for the SF scaffolds with the smallest diameter of fibres: ~250–300 nm (*p* < 0.001). This trend continued until the end of the culture time. During 14 days of cell culture, for the SF scaffolds with increasing fibre diameter, the proliferation of PHDFs decreased. In vitro studies also revealed significantly increased collagen I and III expression in PHDFs cultured on the SF scaffolds with the smallest ~250 nm diameter of fibres. Ex vivo studies on a human full-thickness skin model proved that SF scaffolds promote re-epithelialization in wound healing compared to untreated wounds. Keratinocyte migration was significantly (*p* < 0.05) increased after one week and two weeks in the wounds treated with SF scaffolds compared to the control wounds without treatment.

A noteworthy study was performed by Millán-Rivero et al. [91], who examined SF electrospun scaffolds loaded with human umbilical cord Wharton’s jelly mesenchymal stem cells (Wj-MSCs-SF) and their influence on full-thickness wound healing in hairless SKH1 mice. In this study, control wounds were covered with the commercial dressing Linitul^®^ and with the SF scaffolds. Histological examination showed that wound healing was comparable in the Linitul^®^ and SF scaffold groups. The wounds treated with the Wj-MSCs-SF caused a delay in the wound healing process, but inflammatory infiltration with polymorphonuclear neutrophils (PMN) and macrophages was significantly reduced compared to the SF scaffold group. Infiltration with T CD3+ cells was also decreased in the Wj-MSCs-SF group compared to the SF scaffold group. Furthermore, groups treated with SF scaffolds alone and with the addition of Wj-MSCs in the first two weeks post-injury had significantly (*p* < 0.001) increased vascular surface compared to the Linitul^®^ group, which is crucial in effective skin regeneration.

Silk sericin can stimulate cell migration, proliferation, and collagen production [30,92], which is especially important during wound healing. Moreover, sericin has antioxidant and antimicrobial activity [93,94]. The following procedure for dressing preparation conducted and described in an article by Yu et al. [18] involves both silk proteins: sericin and fibroin. Silkworm cocoons (SC) were treated in a CaCl_2_-ethanol-H_2_O solution in the preparation process. Partially dissolved cocoons, SC sol-gel film (SCSF), were removed from the solution and prepared for further study at specific time points: 30, 60, 90, and 120 minutes. The anti-bacterial performance of the dressings was examined. The highest inhibition against *Escherichia coli* (23.1 mm) and *Staphylococcus aureus* (20.2 mm) was noticed in the SCSF-90 and was significant (*p* < 0.01, *p* < 0.05) compared to the other SCSF dressings. Furthermore, the authors [18] indicated high treated cell viability and the biocompatibility of SCSF dressings, especially SCSF-90. They compared the SCSF-90 scaffold to Mepitel^®^ (silicon-coated polyamide net) and the blank control with no treatment in the wound healing process in rabbit full-thickness wound models. They showed that SCSF-90 effectively enhanced wound healing and epidermal regeneration and reduced the inflammatory process during treatment. Wounds treated with Mepitel^®^ and control wounds without dressing recovered more slowly, with non-uniform thickness of regenerated skin and intense inflammatory infiltration.

Sirong He et al. [95] investigated heparinized SF hydrogels containing FGF1 (acidic fibroblast growth factor 1) and their influence on full-thickness wound healing in Sprague–Dawley rats. In vitro results showed that SF hydrogels with and without FGF1 promoted L929 cell proliferation and migration compared to the untreated blank group. In the in vivo study, the animals were divided into four groups treated with a different dressing: heparinized SF hydrogel, heparinized SF hydrogel with FGF1, commercial chitosan dressing (positive control), and the untreated blank group (3M^TM^ Tegaderm^TM^). The experiment revealed that all kinds of dressings significantly increased wound healing compared to the blank group with no treatment. However, on day 14, wound size was the smallest for the SF dressing containing FGF1 (*p* < 0.001).

A valuable addition to SF-based dressings is chitosan. Chitosan is a biocompatible and biodegradable polysaccharide, presenting antimicrobial and haemostatic activity. More importantly, chitosan also accelerates wound healing [96]. Guang et al. [88] examined a chitosan/SF (CS/SF) composite scaffold in skin wound healing in Sprague–Dawley rats. In vitro examination revealed antimicrobial properties of this dressing after 24 h of a culture with the inhibition diameter against *E. coli* 31 mm, *S. aureus* 14 mm, *Pseudomonas aeruginosa* 22 mm, and *Monilia albicans* 11 mm. Scaffolds made of SF alone did not cause inhibition of microbial growth. In vivo study of a CS/SF composite scaffold on Sprague-Dawley rats showed promotion of skin wound healing without signs of infection and completely healed skin injuries after 21 days.

Silver nanoparticles (AgNPs) can be an influential component of fibroin dressings due to their broad antimicrobial properties [97]. Patil et al. [85] investigated nanosilver-loaded SF (NSF) wound healing activity in male Wistar rats. The authors compared NSF gel with optimized SF gel, silver nanoparticulate gel, Carbopol gel, a positive control (Soframycin gel), and a negative control (untreated wounds). After 15 days of the experiment, the wounds treated with NSF gel had the highest healing rate compared to the other groups and were significant (*p* < 0.05) compared to the Soframycin group.

### 6.2. SF-Based Dressings and Their Use in Burn Wound Healing

Some of the most common skin injuries are burn wounds. They can be caused by abrasion, heat, radiation, chemical agents, electricity, or even cold. Burn wounds are classified depending on the depth of the injury. First-degree burns are characterized by a superficial burn of the epidermis with severe pain and redness without blisters and leaving no scar. Blisters and exudation characterize second-degree burns. Depending on the depth of the wound, this degree is further divided into superficial partial-thickness burns (2A) affecting the epidermis and superficial part of the dermis and deep partial-thickness burns (2B) affecting the epidermis and full-thickness dermis. The advanced degree of burn wounds is third-degree burns, which are injuries to the entire thickness of the skin, including hypodermis [98,99,100,101,102]. There is also a fourth degree of burns involving muscles, tendons, and bones. Such deep burns often lead to the loss of the burned body part [98,101,102]. Treatment of third- and fourth-degree skin burns are mainly based on surgery. However, the treatment of first-degree and superficial partial-thickness burns (2A) does not require surgical procedures and can rely on wound care and medical dressings [98,101,102]. In this context, SF dressings may be an effective treatment option.

Hyung Woo Ju et al. [68] examined an electrospun SF nanomatrix as a potential dressing in a second-degree burn-wound model in Sprague–Dawley rats. The SF nanomatrix was compared to the commercial dressing Medifoam^®^ (polyurethane hydrocelluar dressing foam) and control wounds with medical gauze. After seven days of treatment, wounds dressed with the SF nanomatrix were smaller than wounds treated with the Medifoam^®^. After 28 days of the observation period, the wounds treated with the SF nanomatrix decreased to 4% and wounds with Medifoam^®^ to 8%, while the control wounds with medical gauze decreased only to 18% of their previous size. Histological examination revealed that the SF nanomatrix caused faster skin regeneration and re-epithelialization than the control wounds. Furthermore, the results presented SF immunomodulation activity during wound healing. In the wounds treated with the SF nanomatrix, there was a significantly lower expression of pro-inflammatory IL-1α and IL-6 compared to the Medifoam^®^ and control group on day 7. Expression of anti-inflammatory interleukin 10 (IL-10) was elevated on the 14th and 21st days of observation in wounds treated with the SF nanomatrix compared to the other groups.

Another study was performed by Guan et al. [103] on an SF hydrogel in treating second-degree burns in mice, comparing it to Purilon gel. In vitro studies showed significantly decreased apoptosis rate, higher cell proliferation, and increased cell migration distance in the SF hydrogel groups compared to the Purilon gel and blank group. In vivo study showed that wounds treated with the SF hydrogel healed significantly faster than the Purilon-gel-treated group and the control group (without treatment). Histological examination also showed enhanced re-epithelialisation in the SF-hydrogel-treated group.

A noteworthy study performed by Chouhan et al. [104] examined an SF hydrogel and its influence on skin regeneration in third-degree burn wounds in Wistar albino rats. The SF hydrogel significantly accelerated wound healing compared to untreated wounds. Histological examination showed early development of granulation tissue and rapid re-epithelialisation in treated wounds. By day 14 post-treatment, the wound area in the SF hydrogel-treated wounds was 40%, while the area of untreated wounds was 80%, which represented significantly (*p* ≤ 0.01) accelerated wound healing in the treated wounds compared to the control wounds. Furthermore, SF-treated wounds had higher vessel density compared to untreated wounds. In addition, treatment with the SF dressing revealed increased expression of CD163, a marker of the M2 type of macrophage. It means that the SF-treated wounds were characterized by the infiltration of M2 macrophages, which have a beneficial effect on wound healing by enhancing skin remodelling and regeneration [105].

### 6.3. SF-Based Dressings and Their Use in Diabetic Wound Healing

Diabetes mellitus (DM) is a metabolic disease that can lead to neuropathy and microangiopathy. Peripheral neuropathy causes impaired pain sensation. Due to that, patients often suffer foot deformations or skin irritations and injuries, especially in the distal parts of the body. Furthermore, microangiopathy promotes poor blood flow in the vessels, leading to insufficient delivery of oxygen and nutrients to regenerating tissue. In addition, hyperglycaemia in skin wound healing leads to prolonged inflammatory response, macrophage dysfunction, impaired angiogenesis, and keratinocyte and fibroblast dysfunction [36,106,107,108,109]. All of these factors make skin wound healing in diabetic patients difficult. For this reason, it is crucial to examine new dressings on diabetic models to increase the effectiveness of treatment.

In this regard, SF biomaterials are promising. Li et al. [110] analysed two SF dressings: an SF sponge dressing and an insulin-loaded SF sponge dressing. The authors examined the influence of both dressings on full-thickness wound healing in diabetic Sprague–Dawley rats. After one week of treatment, wound healing in the SF dressings with and without insulin was accelerated compared to untreated wounds. After two weeks, the insulin-loaded SF sponge dressing significantly (*p* < 0.05) increased the wound closure rate compared to the SF sponge dressing and untreated wounds, but the SF dressing without insulin still enhanced wound healing. After three weeks, the wound closure rate was similar in all wounds, but the insulin-loaded SF sponge dressing was superior to the others. In addition, after one and two weeks, the insulin-loaded SF dressing significantly (*p* < 0.05) promoted CD31 expression in the treated wounds compared to the other two groups, indicating its vascularisation-promotion activity.

A similar study on male diabetic Sprague–Dawley rats focused on an insulin-containing SF sponge (INS) dressing was conducted by Yang et al. [62]. The authors compared it to an SF dressing without insulin and examined the influence of these dressings on full-thickness skin wound healing. The results showed significantly (*p* < 0.01) accelerated wound healing in the INS group on days 5 and 11 compared to the control group dressed with an SF dressing without insulin. Reepithelialisation was also significantly (*p* < 0.01) enhanced in the INS group 5 and 11 days post-wounding. Furthermore, the study confirmed the significantly (*p* < 0.01) elevated expression of HIF-1α and its downstream effectors VEGF-A, collagen type I (Col I), and collagen type III (Col III) in the INS-treated group on the 5th day compared to the control group. It indicates the molecular activity of the INS dressing, which resulted in increased neovascular promotion and collagen deposition in the treated wounds.

Another study on diabetic models was performed by Liu et al. [111], who compared three SF-based dressings and their influence on full-thickness wound healing in diabetic Sprague–Dawley rats. They analysed SF scaffolds with neurotensin (NT)—NT/SF, SF scaffolds with gelatine microspheres (GMs) impregnated with the NT—NT/GMs/SF, and SF dressings alone—SF. All these dressings influenced the wound healing process. The most significant wound reduction was found in groups treated with both kinds of dressings containing neurotensin. Histological examination proved macroscopic observations, and the superiority of NT/GMs/SF dressings in wound healing compared to the other groups. The NT/GMs/SF dressing was characterised by an accelerated healing process and significant inflammatory reduction. Neurotensin has pro-angiogenic and immunomodulatory effects [112], making it a valuable component of SF biomaterials to enhance skin regeneration.

A noteworthy study by Maity et al. [113] focused on three types of SF hydrogels and their influence on full-thickness wound healing in diabetic Wistar rats. In the study, the authors examined an SF hydrogel (SFH), an SF melanin hydrogel (SFMH), and an SF composite hydrogel (SFCH) loaded with antioxidant melanin and anti-inflammatory berberine. All these dressings were compared to the untreated control wound (with cotton gauze only). The in vitro studies confirmed the antioxidant properties of SFCH and SFMH dressings, which are especially important during wound healing. Antibacterial activity against *S. aureus* was noticed in the SFCH when SFH and SFMH did not show such activity. The in vitro studies also confirmed higher NIH3T3 cell migration in the SFH, SFMH, and SFCH (the most significant) solutions than in untreated control cells. In vivo studies on diabetic rats showed a higher wound closure rate in all examined hydrogels compared to the untreated wounds at all time points. The most effective wound healing was presented by SFCH, with complete wound contraction in 11 days. Wounds treated with SFH and SFMH completely healed on days 16 and 15, respectively, while untreated control wounds closed on the 18th day of the experiment. Microscopic studies showed the highest re-epithelialization rate, collagen deposition, and vascular density and the lowest epithelial gaps in wounds treated with SFCH.

Navone et al. [114] examined the wound healing effects of electrospun nanofibrous SF patches cellularised with human adipose-derived mesenchymal stromal cells (Ad-MSCs-SF) and decellularized (D-Ad-MSCs-SF) patches in Lepr db/db male diabetic mice. These dressings were compared to the control group with no dressing and positive control with SF only. The in vitro study presented significantly increased HUVEC migration after 24 h and 48 h for Ad-MSCs-SF (*p* < 0.05 and *p* < 0.01) and D-Ad-MSCs-SF (*p* < 0.01) compared to the SF patches. The in vivo results showed the most significant (*p* < 0.05) wound healing on days 3 and 10 for the Ad-MSCs-SF and D-Ad-MSCs-SF groups, with almost complete wound healing on day 10 post-wounding compared to the control group, where wound treatment was delayed up to 15–17 days. Furthermore, on day 7, the Ad-MSCs-SF group noticed significantly increased (*p* < 0.01) expression of VEGFA and other genes involved in angiogenesis compared to the SF group, which is crucial in effective wound healing.

### 6.4. Clinical Study with SF-Based Dressings in Skin Wound Healing

The results of the above-described studies are promising. They encourage conducting SF dressing trials in human skin wound healing. Unfortunately, data on human studies are currently limited. However, Zhang et al. [17] conducted a clinical randomized, single-blind trial on seventy-one patients. The study compared a prepared SF film with the commercial dressing Sidaiyi (SF sponge-silicone, two-layered scaffold) in skin wound healing. The results showed that the SF film significantly (*p* = 0.015) accelerated wound healing compared to the commercial dressing. Furthermore, during the study there were three inflammation and three exudate cases in wounds treated with Sidaiyi. In contrast, there were not any cases of exudate and only one case of inflammation in the SF film-treated wounds. The authors also conducted pre-clinical trials on rabbits and pigs and compared the SF film with Sidaiyi, Suprathel (skin substitute), and an untreated blank group in full-thickness skin wound healing. These studies demonstrated the effectiveness and superiority of an SF film over commercial dressings and untreated groups in full-thickness skin wound healing.

All of the above studies are summarized in Table 1, regarding the type of SF dressing, control dressings, examined dressings, and the rate of skin healing considering the *p*-value.

## 7. Conclusions

SF is a biocompatible and biodegradable biomaterial. Thanks to its properties, it has a wide range of medical applications, including wound healing. Once fibroin is isolated from silk cocoons, the SF solution can be used to make various types of dressings. Acting on multiple signalling pathways stimulates cell growth and migration, promotes pro-angiogenic action, and significantly accelerates skin wound healing. In addition, SF-based dressings form a scaffold that can be loaded with additional substances that enhance tissue regeneration, reduce inflammation, or have antimicrobial activity. SF-based dressings have been tested not only on full-thickness skin wounds but also on burns and hard-to-heal diabetic wounds, and have shown high efficacy, even compared to commercially available preparations and dressings. Most of the available studies in the literature concern preclinical studies on animal models. Clinical data on the effect of such dressings on wound healing in humans is limited but promising, providing motivation to initiate such studies on patients and analyse them thoroughly.

## Figures and Tables

**Table 1:** Summary of SF biomaterials in skin wound healing.

Author	Type of SF Dressing	Control Dressing	Dressed Wound	Healing Rate (*p*-Value)
[59]	Scaffold	No dressing	Random SF scaffold (RSS)	*p* ≤ 0.01 on days 3 and 21, *p* ≤ 0.05 on day 14, *p* ≤ 0.001 on day 21
			Aligned SF scaffold (ASS)	*p* ≤ 0.05 on days 3, 7, 14 *p* ≤ 0.001 on day 21
[90]	Electrospun membrane	3M^TM^ Tegaderm^TM^	PDA-modified electrospun SF membrane (PESF)	*p* < 0.001
			Electrospun SF membrane (ESF)	Not significant
[60]	Electrospun scaffold	No dressing	Electrospun SF scaffold	*p* < 0.05
[91]	Electrospun scaffold	Linitul^®^	Electrospun SF scaffolds cellularized with human Wharton’s jelly MSCs (Wj-MSCs-SF)	-
[18]	Sol-gel film	No dressing (control group), Mepitel^®^ (positive control)	Silkworm cocoonss ol-gel film (SCSF-90)	*p* < 0.05 on day 5 *p* < 0.01 on days 10 and 15, compared to the control group
[95]	Hydrogel	3M^TM^ Tegaderm^TM^ (control group), commercial chitosan dressing (positive control)	Heparinized SF hydrogel	-
			Heparinized SF hydrogel with FGF1 (SF + FGF)	*p* < 0.001 on day 14, compared to the control group
[88]	Scaffold	-	Chitosan/SF (CS/SF) composite scaffold	-
[85]	Hydrogel	Soframycin gel (positive control)	Nanosilver-loaded SF (NSF) gel	*p* < 0.05
[68]	Electrospun nanomatrix	Medical gauze (control group), Medifoam^®^ (positive control)	Electrospun SF nanomatrix	-
[103]	Hydrogel	No dressing (control group), Purilon gel (positive control)	SF hydrogel	*p* < 0.05 on days 9 and 12 compared to the Purilon gel
[104]	Hydrogel	Tegaderm^TM^ film (control group), Col gel (positive control)	SF hydrogel	*p* ≤ 0.01 compared to the control group
[110]	Sponge	Gauze	SF sponge dressing	-
			Insulin-loaded SF sponge dressing	*p* < 0.05 after 1 and 2 weeks compared to the other groups
[62]	Sponge	SF sponge dressing	Insulin-loaded SF sponge dressing	*p* < 0.01 on days 5 and 11
[111]	Scaffold	No dressing	SF	-
			NT/SF	-
			NT/GMs/SF	*p* < 0.05 on days 14, 21, and 28
[113]	Hydrogel	Gauze (control group), SFH (4% SF; positive control)	SFMH (4% SF with 0.01% melanin)	-
			SFCH (4% SF, 0.01% melanin, and 0.1% of berberine)	-
[114]	Electrospun scaffold	No dressing (control group), SF scaffold (positive control)	SF patches cellularised with human adipose-derived mesenchymal stromal cells (Ad-MSCs-SF)	*p* < 0.05, on days 3 and 10 compared to the control group
			Decellularized (D-Ad-MSCs-SF)	*p* < 0.05, on days 3 and 10 compared to the control group
[17], clinical study	Film	Commercial dressing Sidaiyi (SF sponge-silicone, two-layered scaffold)	SF film	*p* = 0.015

## Data Availability

Not applicable.
